# Pharmacological enhancement of cholinergic neurotransmission alleviates neuroinflammation and improves functional outcomes in a triple transgenic mouse model of Alzheimer’s disease

**DOI:** 10.3389/fphar.2024.1386224

**Published:** 2024-03-26

**Authors:** Antonio Munafò, Anna Flavia Cantone, Giulia Di Benedetto, Sebastiano Alfio Torrisi, Chiara Burgaletto, Carlo Maria Bellanca, Gabriella Gaudio, Giuseppe Broggi, Rosario Caltabiano, Gian Marco Leggio, Renato Bernardini, Giuseppina Cantarella

**Affiliations:** ^1^ Department of Biomedical and Biotechnological Sciences, Section of Pharmacology, University of Catania, Catania, Italy; ^2^ Clinical Toxicology Unit, University Hospital of Catania, Catania, Italy; ^3^ Department of Medical, Surgical Sciences and Advanced Technologies “G.F. Ingrassia”, Anatomic Pathology, University of Catania, Catania, Italy

**Keywords:** amyloid plaques, choline alphoscerate, glial cells, memory, mouse brain, neurodegeneration, synapses

## Abstract

**Introduction:** Alzheimer’s disease (AD) is the most common neurodegenerative disorder affecting the elderly population worldwide. Due to the multifactorial nature of the disease, involving impairment of cholinergic neurotransmission and immune system, previous attempts to find effective treatments have faced challenges.

**Methods:** In such scenario, we attempted to investigate the effects of alpha-glyceryl-phosphoryl-choline (α-GPC), a cholinomimetic molecule, on neuroinflammation and memory outcome in the triple transgenic mouse model of AD (3xTg-AD). Mice were enrolled at 4 months of age, treated orally with α-GPC dissolved in drinking water at a concentration resulting in an average daily dose of 100 mg/kg for 8 months and sacrificed at 12 months of age. Thereafter, inflammatory markers, as well as cognitive parameters, were measured.

**Results:** Chronic α-GPC treatment reduced accumulation of amyloid deposits and led to a substantial re-balance of the inflammatory response of resident innate immune cells, astrocytes and microglia. Specifically, fluorescent immunohistochemistry and Western blot analysis showed that α-GPC contributed to reduction of cortical and hippocampal reactive astrocytes and pro-inflammatory microglia, concurrently increasing the expression of anti-inflammatory molecules. Whereas α-GPC beneficially affect the synaptic marker synaptophysin in the hippocampus. Furthermore, we observed that α-GPC was effective in restoring cognitive dysfunction, as measured by the Novel Object Recognition test, wherein 3xTg-AD mice treated with α-GPC significantly spent more time exploring the novel object compared to 3xTg-AD untreated mice.

**Discussion:** In conclusion, chronic treatment with α-GPC exhibited a significant anti-inflammatory activity and sustained the key function of hippocampal synapses, crucial for the maintenance of a regular cognitive status. In light of our results, we suggest that α-GPC could be exploited as a promising therapeutic approach in early phases of AD.

## 1 Introduction

Alzheimer’s disease (AD) is a progressive neurodegenerative disorder representing the major cause of dementia worldwide, characterized by an irreversible decline in episodic memory and a general deterioration in overall cognitive ability (Knopman et al., 2021).

So far, only treatments that address symptoms with limited disease-modifying properties are available for Alzheimer’s disease (AD) (Cummings et al., 2023). This underscores the substantial unmet needs within the AD landscape. Despite numerous attempts to introduce innovative neuroprotective treatments, the persistent lack of satisfactory solutions remains a challenge (Kim, 2015).

AD is pathologically underpinned by accumulation of extracellular amyloid-β (Aβ) plaques, intracellular neurofibrillary tangles of hyperphosphorylated tau protein, resulting in synaptic and neuronal loss as well as marked neuroinflammation (Holtzman et al., 2011; Burgaletto et al., 2020).

Selective degeneration of cholinergic neurons of basal forebrain and subsequent dysfunction of cholinergic transmission have long been deemed as driving factors for disease development and, therefore, have steadily directed the main therapeutic efforts in the drug discovery process (Francis et al., 1999). Impairment of forebrain cholinergic neurons, regulating innate immune responses and inflammation, leads to cholinergic dysfunction (Canu et al., 2017). Cholinergic precursors have been among the initial therapeutic strategies attempting to counteract cholinergic impairment and to relieve cognitive decline occurring in dementia disorders. Among these, choline alphoscerate, also known as alpha-glyceryl-phosphoryl-choline (α-GPC), is considered one of the most suitable sources of choline (Parnetti et al., 2001; Traini et al., 2013; Sagaro et al., 2023). Despite its presence in the pharmaceutical market since 1987, α-GPC experienced a decline in interest following the introduction of cholinesterase inhibitors. Nevertheless, the last 10 years have witnessed a resurgence of interest in this choline-containing phospholipid, evident in numerous pre-clinical and clinical studies (Traini et al., 2013; Traini et al., 2020; Roy et al., 2022; Sagaro et al., 2023). Indeed, α-GPC, encompassing choline in its structure, seems to have a significant effect in enhancing acetylcholine (Ach) synthesis and release, due to its ability to cross the blood-brain-barrier (BBB) (Parnetti et al., 2007). Several preclinical studies have shown that α-GPC promotes learning and memory in experimental aging models (Lopez et al., 1991; Traini et al., 2013; Matsumura et al., 2015) and it has been proven effective in reversing mnemonic deficits induced by scopolamine administration (Sigala et al., 1992). Although beneficial effects of α-GPC have been extensively reported in experimental models, only sparse research have assessed the mechanisms underlying such effects. Recently, *in vitro* experiments have revealed the α-GPC protective role upon Aβ toxicity by setting into motion neurotrophins-signaling pathways (Burgaletto et al., 2021) and by counteracting inflammation associated with AD (Cantone et al., 2024). Notably, the heightening of cholinergic transmission contribute to suppression of glial pro-inflammatory cytokines production, as well as the enhancement of Aβ clearance, synaptic plasticity and memory (Buckingham et al., 2009). This intricate interplay between neuroinflammation and cholinergic transmission responses underscores the complexity of AD pathogenesis and highlights potential therapeutic targets aimed at mitigating disease progression.

**FIGURE 1:**
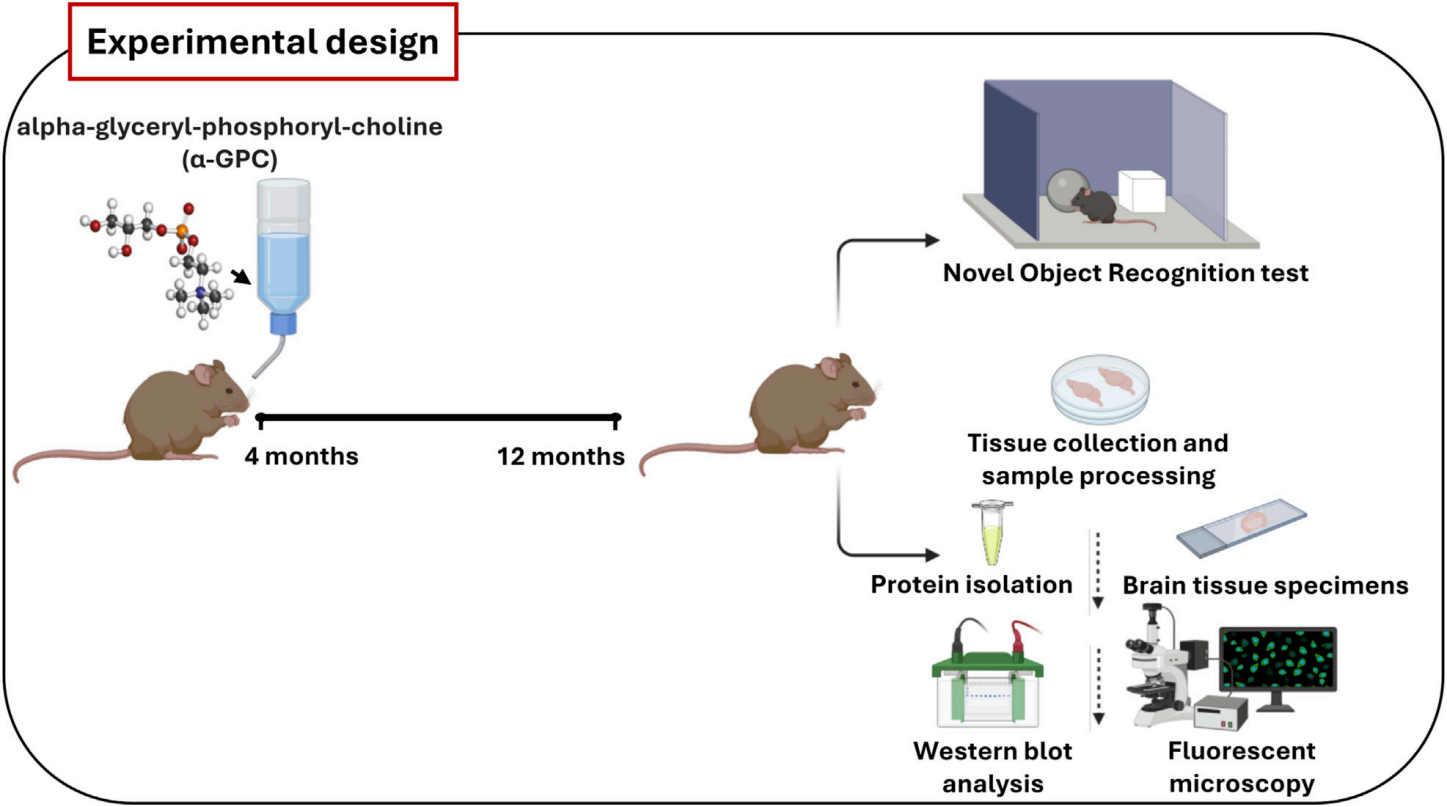
Schematic representation of the main steps of *in vivo* experiments. Wild-type (WT) and 3xTg-AD mice were treated orally with vehicle or α-GPC (100 mg/kg/day) dissolved in drinking water for 8 months. At 12 months of age, animals performed Novel Object Recognition test or were sacrificed and tissues were collected to perform protein analysis.

In such scenario, the primary aim of our study was to explore, for the first time, whether the chronic treatment with α-GPC could contribute to an immune rebalance, and whether this phenomenon correlated with an improvement of the cognitive outcome in AD. To address this objective, we employed the 3xTg-AD mouse model, which accurately replicates AD pathology and cognitive decline, allowing us to assess, through methods such as fluorescent immunohistochemistry, Western blot analysis and behavioral test, α-GPC’s impact on neuroinflammation, synaptic function, and cognitive performance (Figure 1).

## 2 Materials and methods

### 2.1 Animals

Male 3xTg-AD (Oddo et al., 2003) mice [B6129-Psen1tm1MpmTg (APPSwe, tauP30L) 1Lfa/J] [14] and wild-type mice (B6129SF2/J) were purchased from Jackson Laboratories. The 3xTg-AD, overexpressing mutant APP (APPSwe), PSEN1 (PS1M146V), and hyperphosphorylated tau (tauP301L), were originally generated by co-injecting two independent transgene constructs encoding human APPSwe and tauP301L (4R/0 N) (controlled by murine Thy1.2 regulatory elements) in single-cell embryos harvested from mutant homozygous PS1M146V knock-in mice. Age-matched wild-type mice of mixed genetic background 129/C57BL6 were used as controls. The mice were maintained on a 12-h light/dark cycle in temperature and humidity-controlled rooms, and food and water were available *ad libitum*. Animal experiments were conducted in accordance with the recommendations in the Guide for the Care and Use of Laboratory Animals (FELASA). The animal study protocol was approved by the Italian Ministry of Health (authorization n.552/2020-PR) and conducted following the European Community directive guidelines for the use of animals in laboratory (2010/63/EU) and the Italian law (D.Lgs. 26/2014).

### 2.2 Drug administration and experimental groups

Alpha-glyceryl-phosphoryl-choline (α-GPC) was purchased from Italfarmaco, Milano, Italy. Twenty-two 3xTg-AD and twenty-two wild-type mice were enrolled at 4 months of age and four study groups were used: (i) wild-type plus vehicle (water) (n = 11); (ii) wild-type plus 100 mg/kg/day α-GPC (n = 11); (iii) 3xTg-AD plus vehicle (water) (n = 11); and (iv) 3xTg-AD plus 100 mg/kg/day α-GPC (n = 11); (mouse weight = 25 ± 5 g). Animals belonging to the second and fourth group received α-GPC dissolved in drinking water at a concentration resulting in an average daily dose of 100 mg/kg according to the procedure detailed elsewhere (Amenta et al., 1991). Animals were sacrificed after 8 months of treatment via CO2 inhalation.

### 2.3 Protein extraction

Brain samples of 3xTg-AD and age-matched wild-type mice were dissected in ice-cold Hank’s balanced salt solution (HBSS: 137 mM NaCl, 5.4 mM KCl, 0.45 mM KH2PO4, 0.34 mM Na2HPO4, 4 mM, NaHCO3, 5 mM glucose; pH 7.4) and then stored at −80 °C, until use. Brain tissues were lysed in a lysis buffer containing 150 manacle, 50 mM Tris–HCl (pH 7.5), 5 mM EDTA, 1 mM Na3VO4, 30 mM sodium pyrophosphate, 50 mM NaF, 1 mM acid phenyl-methyl-sulphonyl- fluoride, 5 mg/mL aprotinin, 2 mg/mL leupeptin, 1 mg/mL pepstatin, 10% glycerol, and 0.2% TritonTM X-100. The homogenates were then centrifuged at 14000 rpm for 10 min at 4°C. The protein concentration of the supernatant was determined by the Bradford method (Bradford, 1976).

### 2.4 Western blot analysis

Equal amounts of proteins (50 µg) were separated by 8%–15% SDS- PAGE gels and transferred onto Hybond ECL nitrocellulose membranes (10600003, Amersham Life Science, Buckinghamshire, UK). The membranes were blocked with 5% non-fat dry milk in PBST for 1 h at RT and were then probed overnight at 4°C on orbital shaker with the following appropriate primary antibodies: mouse anti-β-amyloid (1:500, SIG-39320, Covance, Princeton, New Jersey, United States of America) mouse anti-synaptophysin (1:500, ab8049, Abcam, Cambridge, UK), goat anti-Iba1 (1:1000, NB100-1028, Novus Biologicals, Littleton, Colorado), mouse anti-GFAP (1:500, sc-166458, Santa Cruz Biotechnology Inc., Santa Cruz, CA, United States of America), rabbit anti-iNOS (1:500, sc-7271, Santa Cruz Biotechnology Inc.), rabbit anti-IL-10 (1:200, 250713, Abbiotec, San Diego, CA, United States of America;), rabbit anti-TNF-α (1:1000, NB600-587, Novus Biologicals). Mouse anti-β-Actin (1:1000, sc-47778, Santa Cruz Biotechnology Inc.) primary antibody was used as an internal control to validate the right amount of protein loaded in the gels. Then the membranes were washed with PBS-T and probed with the appropriate horseradish peroxidase-conjugated secondary antibodes (sheep anti-mouse NXA931V, Amersham Life Science or a donkey anti-rabbit NA934V, Amersham Life Science or a mouse anti-goat sc-2354, Santa Cruz Biotechnology Inc.) for 1 h at room temperature in 5% non-fat dry milk. After washing with PBS-T, protein bands were visualized by enhanced chemiluminescence (Thermo Fisher Scientific, Inc, Massachussets, United States of America) and scanned with the iBright FL1500 Imaging System (Thermo Fisher Scientific). Densitometric analysis of band intensity was performed with the aid of ImageJ software version 1.53v (developed by NIH, freeware, available online: https://imagej.nih.gov/ij/, accessed on 6 November 2023).

### 2.5 Immunofluorescence

Mice were deeply anesthetized and intracardially perfused with ice-cold 4% paraformaldehyde (PFA). Collected brain tissue specimens were fixed overnight in 10% neutral-buffered formalin (Bio-Optica). After overnight washing, they were dehydrated in graded ethanol and paraffin-embedded taking care to preserve their anatomical orientation. Tissue sections of 5 µm were then cut and mounted on silanized glass slides and air dried. To remove the paraffin, slides were immersed in xylene two times, for 10 min each; rehydrated with graded ethanol, 100%, 95%, 70%, and 50%, two times for 10 min each; and transferred to distilled water. Antigens were retrieved in sodium citrate buffer (10 mM sodium citrate, 0.05% Tween-20, pH 6.0) by microwave for 10 min, followed by rinsing with distilled water. The slides were then washed in PBS containing 0.025% Tween-20 (PBS-T) twice for 5 min each, blocked in 5% BSA/0.3% PBST for 1 h at room temperature, in humid chamber, and incubated overnight at 4°C with BSA 1% and the following primary antibodies: mouse anti-β-Amyloid (1:200, SIG-39320, Covance), or goat anti-Iba1 antibody (1:100, NB100-1028, Novus Biologicals) or a rabbit anti-IL-10 antibody (1:200, 250713, Abbiotec) or a rabbit anti-GFAP antibody (1:500, Z0334, Dako, Glostrup, Denmark), or a mouse anti-NOS2 antibody (1:250, sc-7271, Santa Cruz) or a rabbit anti-TNFα antibody (1:100, NB600-587, Novus Biologicals) or a mouse anti-synaptophysin antibody (1:100, ab8049, Abcam). Antibodies were applied directly onto sections before overnight slide incubation (4°C) in a humid chamber. For immunopositivity reactions and fluorescence detection, after washing in PBST three times for 5 min each, sections were incubated with the corresponding fluorescent-labeled secondary antibodies in the dark for 1 h at room temperature: Alexa Fluor 546 donkey anti-goat IgG (A11056, Thermo Fisher Scientific, Inc.) or Alexa Fluor 488 donkey anti-rabbit (A21206, Thermo Fisher Scientific) or Alexa Fluor 488 donkey anti-mouse (A21202, Thermo Fisher Scientific) or Alexa Fluor 546 donkey anti-mouse (A10036, Thermo Fisher Scientific). Finally, for staining of nuclei and stabilization of fluorescent signals, slides were covered in mounting medium (F6057, Fluoroshield with DAPI; Sigma-Aldrich, Milan, Italy) and secured with a coverslip. Fluorescence images were captured using a Zeiss Observer. Z1 microscope equipped with the Apotome.2 acquisition system (Zeiss LSM 700, Jena, Germany).

### 2.6 Novel object recognition (NOR) test

The NOR test was performed as previously described with minor modifications (Torrisi et al., 2023). The behavioral test was performed in regularly illuminated (40 ± 1 lux) grey open fields (44 × 44 × 40 cm, cat. no. 47432, Ugo Basile, Gemonio, Italy). The objects were different in shape, color and size (4 × 4 × 4 cm to 6 × 6 × 6 cm). They were fixed to the floor of the apparatus to circumvent displacements during the test. The researchers handled animals on alternate days during the week preceding the stress procedure. Animals were acclimatized to the testing room 1 h before the beginning of the tests. A 2-day pretest was performed to acclimatize mice to the apparatus as well as to prevent neophobia during the test. Mice were placed into the empty apparatus and allowed to freely explore for 15 min on day 1. Mice were instead allowed to explore the apparatus with two objects (different from those eventually used during the test) for 10 min during the day 2. The objects were placed in two corners of the apparatus, 10 cm far from the side walls. The test consisted of one sample phase and one test phase interspersed with 24 h delay in order to assess long-term recognition memory. During the sample phase (day 3), animals were placed in the center of the apparatus and allowed to explore two identical copies of an object for a total of 10 min. During the test phase, mice were allowed to explore for 10 min a copy of the familiar object previously explored in the sample phase, and a novel object never encountered. Mice performing a total exploration of the objects below 5 s were excluded from the analysis. If the long-term recognition memory is intact, mice typically explore more the novel object rather than the familiar object. Cognitive performance during the test session was showed using the discrimination index (DI), calculated using the following formula: [(time spent exploring the novel object–time spent exploring the familiar object)/total exploration time]. The percentage of exploration of each object during the test session were also quantified. Behavioral experiments were carried out, recorded and analyzed by two expert researchers. The exploration of the objects was manually scored by the researchers. Each open field was cleaned with a 20% ethanol solution between sessions to minimize the impact of olfactory cues. A 12 h light/12 h dark cycle with was used. All behavioral experiments were performed during the light phase (9.00 a.m.–4.00 p.m.).

### 2.7 Statistical analysis

Data were analyzed using the analysis of variance (ANOVA), followed by Bonferroni *post hoc* test. Vertical bars are means ± S.E.M. Statistical significance was set at a *p-value <0.05. The graphs and statistical evaluation were made using Graph Pad Prism (Ver. 8, La Jolla, United States).

## 3 Results

### 3.1 α-GPC chronic treatment reduces beta-amyloid plaques formation in 3xTg-AD mice

**FIGURE 2:**
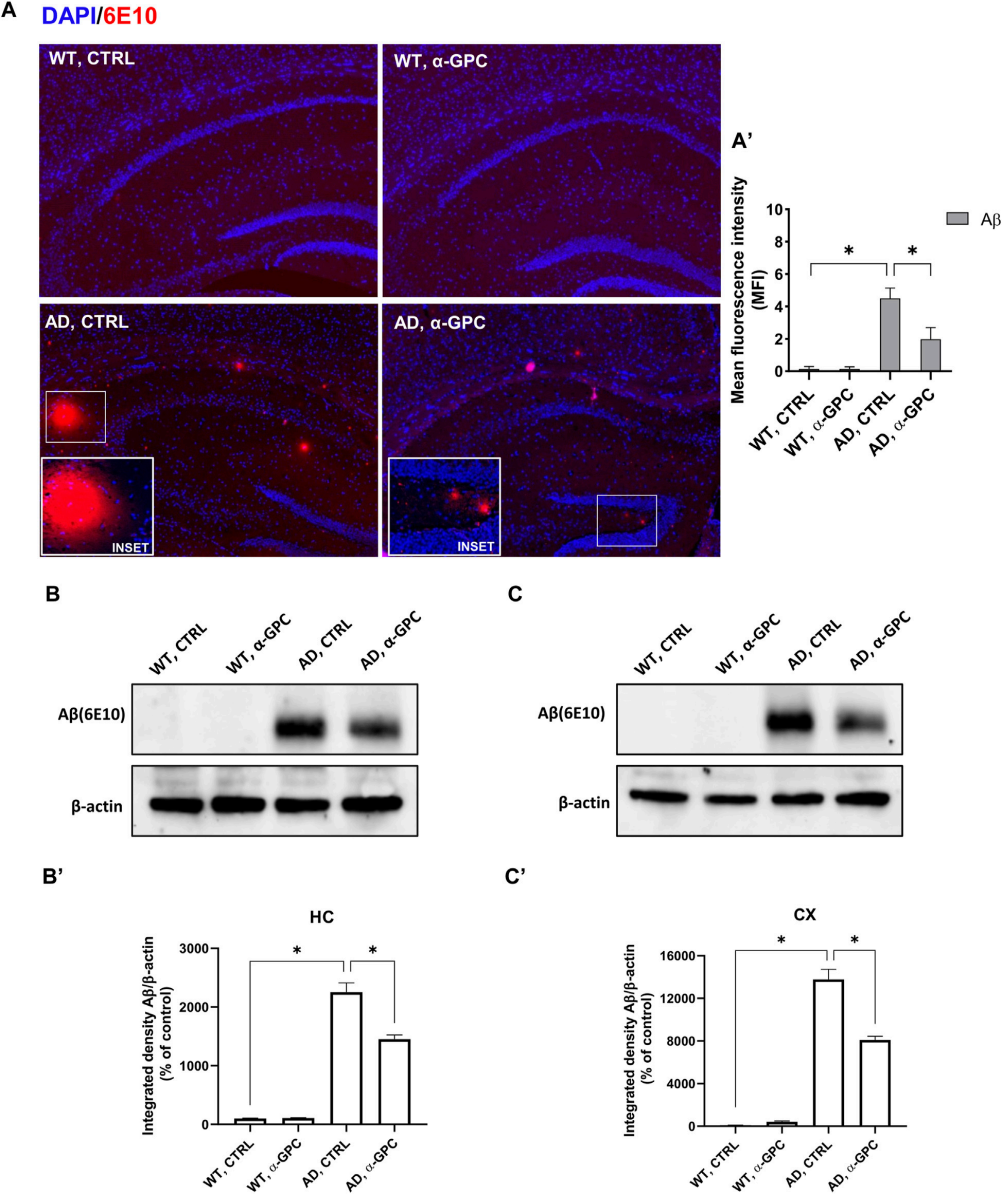
Chronic administration of α-GPC is linked with reduced Aβ-plaques in 3xTg-AD mice. **(A)** Immunohistochemical staining for Aβ-plaques in the brain of wild-type and 3xTg-AD mice, treated either with vehicle or α-GPC. Original magnification 5x; inset 20x. The inserts in photos represent the respective area magnified. **(A′)** Respective mean fluorescence intensity (MFI) analysis of the immunofluorescence signal. Western blot analysis of Aβ protein expression in the hippocampus **(B)** and cortex **(C)** of wild-type and 3xTg-AD mice, following chronic treatment with α-GPC or vehicle and respective densitometric analysis **(B′,C′)** (HC = hippocampus; CX = cortex). Data are expressed as means ± S.E.M. One-way ANOVA and the Bonferroni *post hoc* test were used to determine statistical significance. *p < 0.05. WT: wild-type (n = 3/group); AD: 3xTg-AD mice (n = 3/group).

The most prominent feature of AD is the aggregation of the amyloid-β peptide (Walker, 2020). In order to explore the potential of chronic α-GPC treatment in modulating the formation of Aβ plaques, the expression of Aβ immunopositive deposits was evaluated in brain tissues of 3xTg-AD mice. Immunofluorescence experiments revealed widespread Aβ plaques in the brain of 3xTg-AD untreated mice, while mice that underwent α-GPC chronic treatment exhibited a marked reduction in plaque formation (Figure 2A). Furthermore, Western blot analysis unveiled a significant reduction in hippocampal and cortical expression of amyloid-β in 3xTg-AD animals subjected to chronic α-GPC treatment (Figures 2B, C).

### 3.2 Chronic treatment with α-GPC is associated with reduction of gliosis in 3xTg-AD mice

**FIGURE 3:**
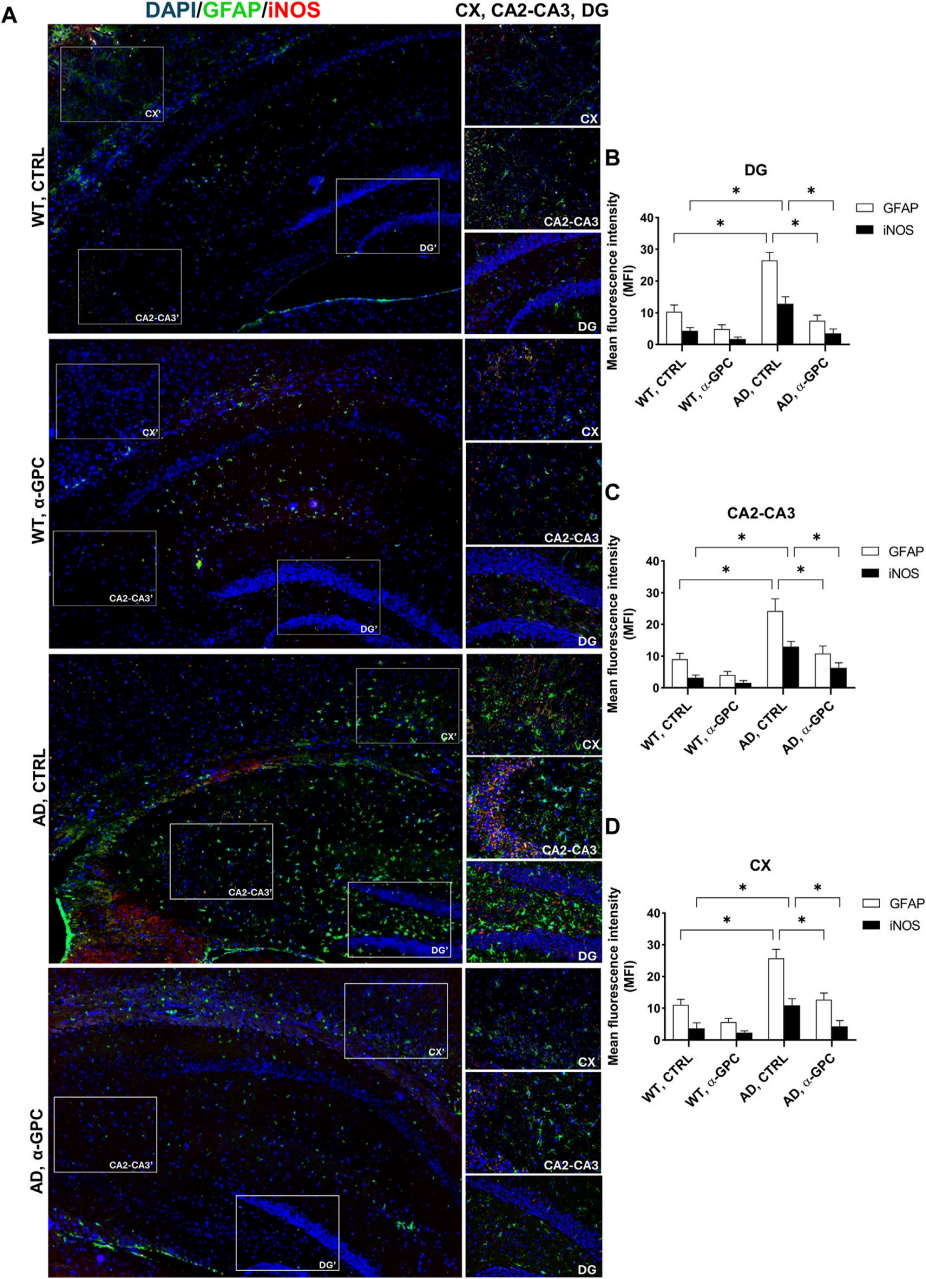
Chronic treatment with α-GPC is associated with reduction of gliosis in 3xTg-AD mice. **(A)** Immunohistochemical staining for GFAP and iNOS in the brain of wild-type and 3xTg-AD mice, treated either with vehicle or α-GPC. Original magnification 5x; inset 20x. The inserts in photos represent the respective areas magnified, DG = dentate gyrus; CA2-CA3 = cornu ammonis 2–3; CX = cortex. **(B,C and D)** Respective mean fluorescence intensity (MFI) analysis of the immunofluorescence signal in the different brain areas. Data are expressed as means ± S.E.M. One-way ANOVA and the Bonferroni *post hoc* test were used to determine statistical significance. *p < 0.05. WT: wild-type (n = 3/group); AD: 3xTg-AD mice (n = 3/group).

**FIGURE 4:**
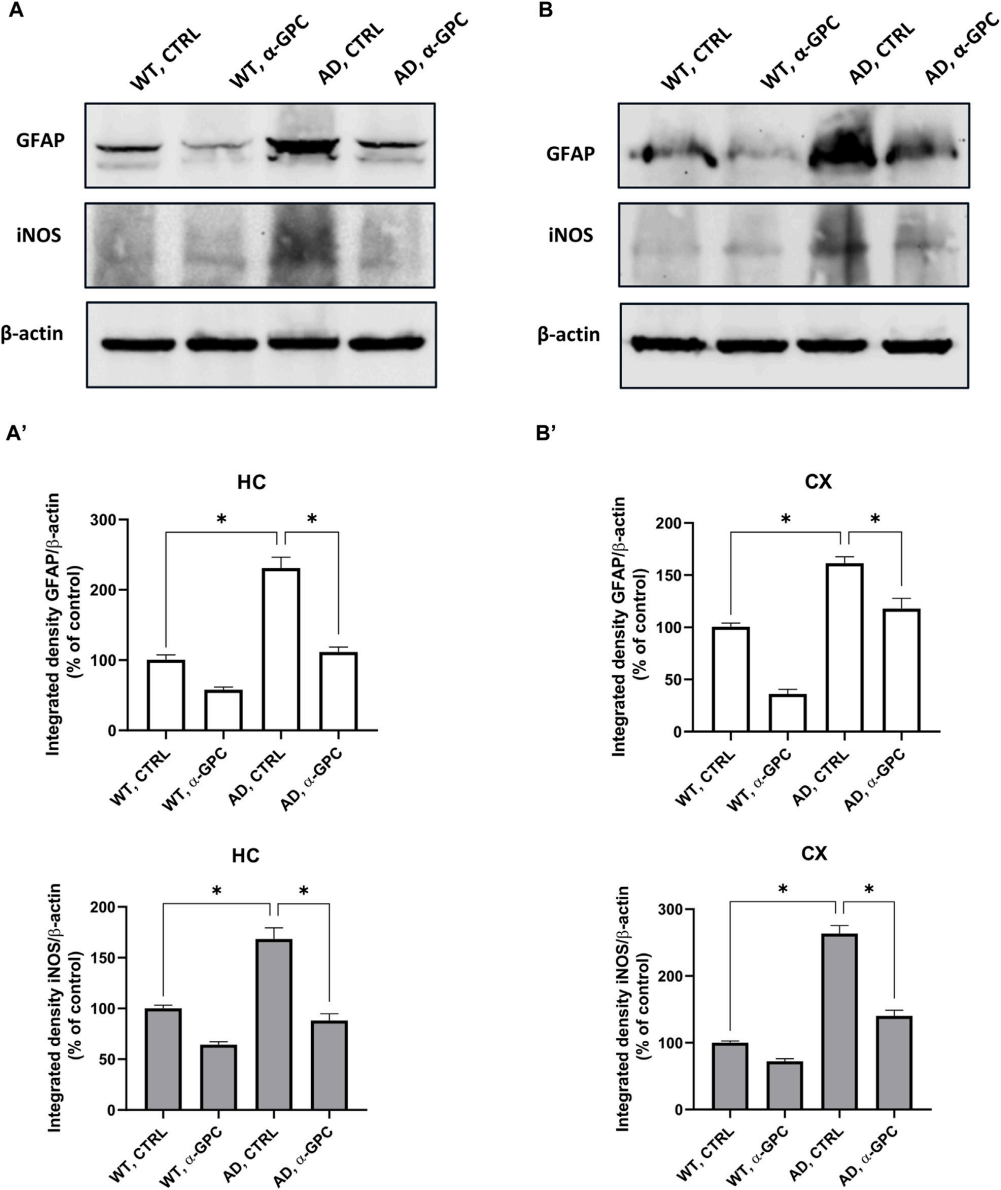
Western blot analysis of GFAP and iNOS protein expression in the hippocampus **(A)** and cortex **(B)** of wild-type and 3xTg-AD mice, following chronic treatment with α-GPC or vehicle and respective densitometric analysis **(A′,B′)** (HC = hippocampus; CX = cortex). Data are expressed as mean ± S.E.M. Differences between groups were considered significant at *p < 0.05 (One-way ANOVA followed by Bonferroni *post hoc* test). WT: wild-type (n = 3/group); AD: 3xTg-AD mice (n = 3/group).

Reactive gliosis is considered a key abnormality in neurodegenerative diseases, representing one of the most important mechanisms in AD neuropathology (De Sousa, 2022). Therefore, with the aim to verify whether chronic treatment with α-GPC could affect reactive gliosis features, expression of GFAP and iNOS (Asiimwe et al., 2016) was evaluated in brain tissues (cortex and hippocampus) of 3xTg-AD mice. Immunofluorescent labeling revealed that 3xTg-AD mice showed a broad astrocytic activation, as demonstrated by the increased expression of GFAP, which co-localized with iNOS, in both hippocampus and cortex, as compared to WT mice (Figure 3). Notably, the expression of both GFAP and iNOS was dramatically decreased in α-GPC treated animals. Consistently with immunohistochemical data, Western blot analysis corroborate the significative reduction of marker expression in α-GPC treated animals in both hippocampus (Figure 4A) and cortex (Figure 4B).

### 3.3 Proinflammatory microglia is blunted by α-GPC chronic treatment in 3xTg-AD mice

**FIGURE 5:**
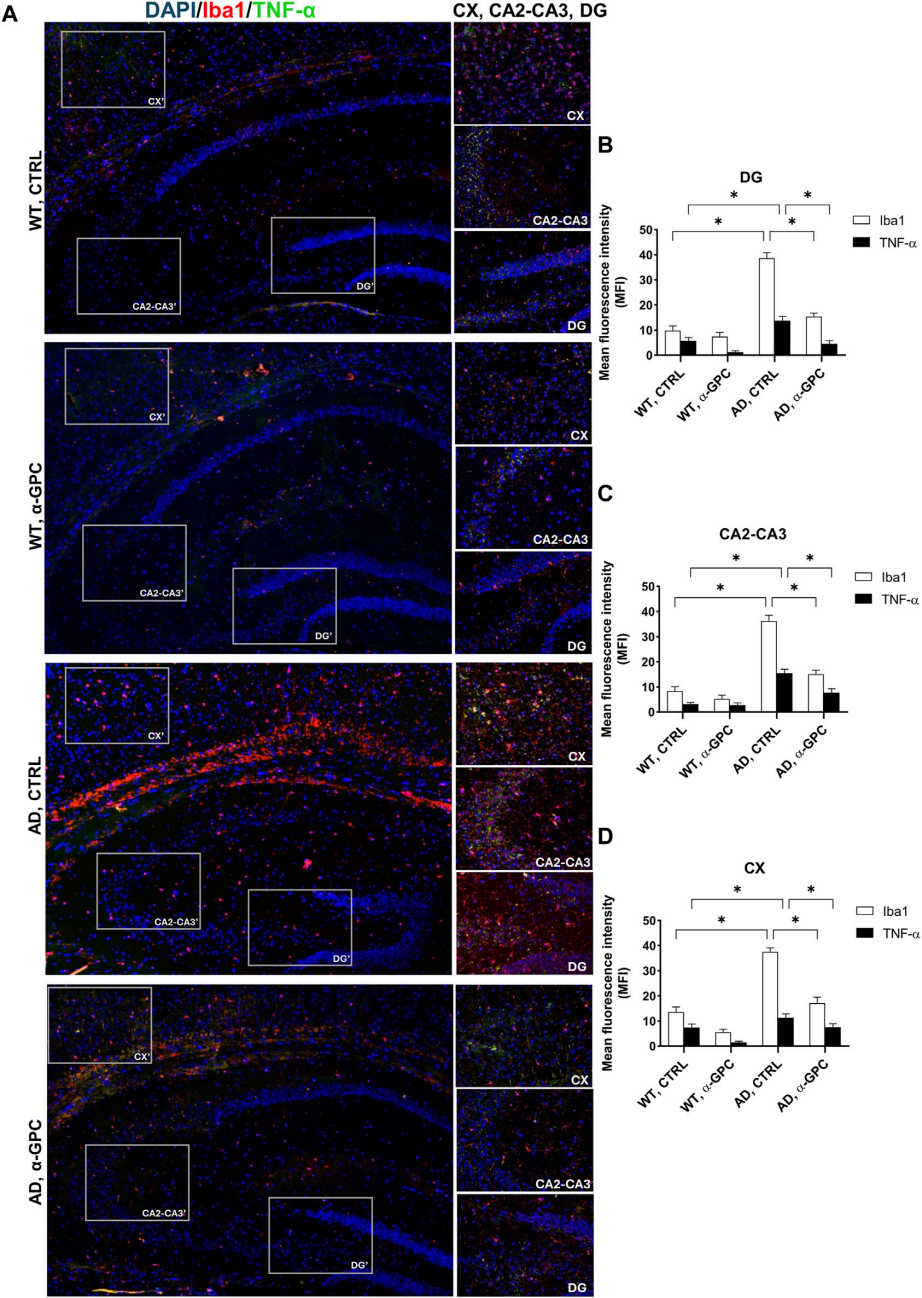
α-GPC reduces the expression of proinflammatory markers in 3xTg-AD mice and blunts microglia activation. **(A)** Immunohistochemical staining for Iba-1 and TNF-α in the brain of wild-type and 3xTg-AD mice, treated with either vehicle or α-GPC. Original magnification, 5x; inset 20x. The inserts in photos represent the respective areas magnified (DG = dentate gyrus; CA2-CA3 = cornu ammonis 2–3; CX = cortex). **(B–D)** Respective mean fluorescence intensity (MFI) analysis of the immunofluorescence signal in the different brain areas. Data are expressed as means ± S.E.M. One-way ANOVA and the Bonferroni *post hoc* test were used to determine statistical significance. *p < 0.05. WT: wild-type (n = 3/group); AD: 3xTg-AD mice (n = 3/group).

Microglia housekeeping functions are essential to maintain brain health (Tremblay et al., 2011). In contrast, chronic activation of microglia, which occurs in AD, causes brain inflammation leading to neuronal death (Wang et al., 2023). To assess the potential of chronic α-GPC treatment in modulating microglia activation, brain tissues were stained with the microglial marker Iba1 and TNF-α, a pro-inflammatory cytokine. Immunofluorescent staining revealed a widespread glia activation in 3xTg-AD mice, as evidenced by the increase in expression of microglial Iba1 co-localized with TNF-α in both hippocampus and cortex. Remarkably, chronic treatment with α-GPC clearly attenuated microgliosis in such brain areas of 3xTg-AD mice (Figure 5). These data were consistent with those obtained by Western blot analysis and, eventually revealed that Iba1-positive cells were significantly decreased in α-GPC treated animals when compared with untreated AD mice. Moreover, Western blot results also demonstrated a substantial decrease in Iba1 and TNF-α expression levels in the hippocampus and cortex of 3xTg-AD mice following α-GPC treatment (Figures 7A, B).

### 3.4 α-GPC chronic treatment switches microglia towards a neuroprotective phenotype in 3xTg-AD mice

**FIGURE 6:**
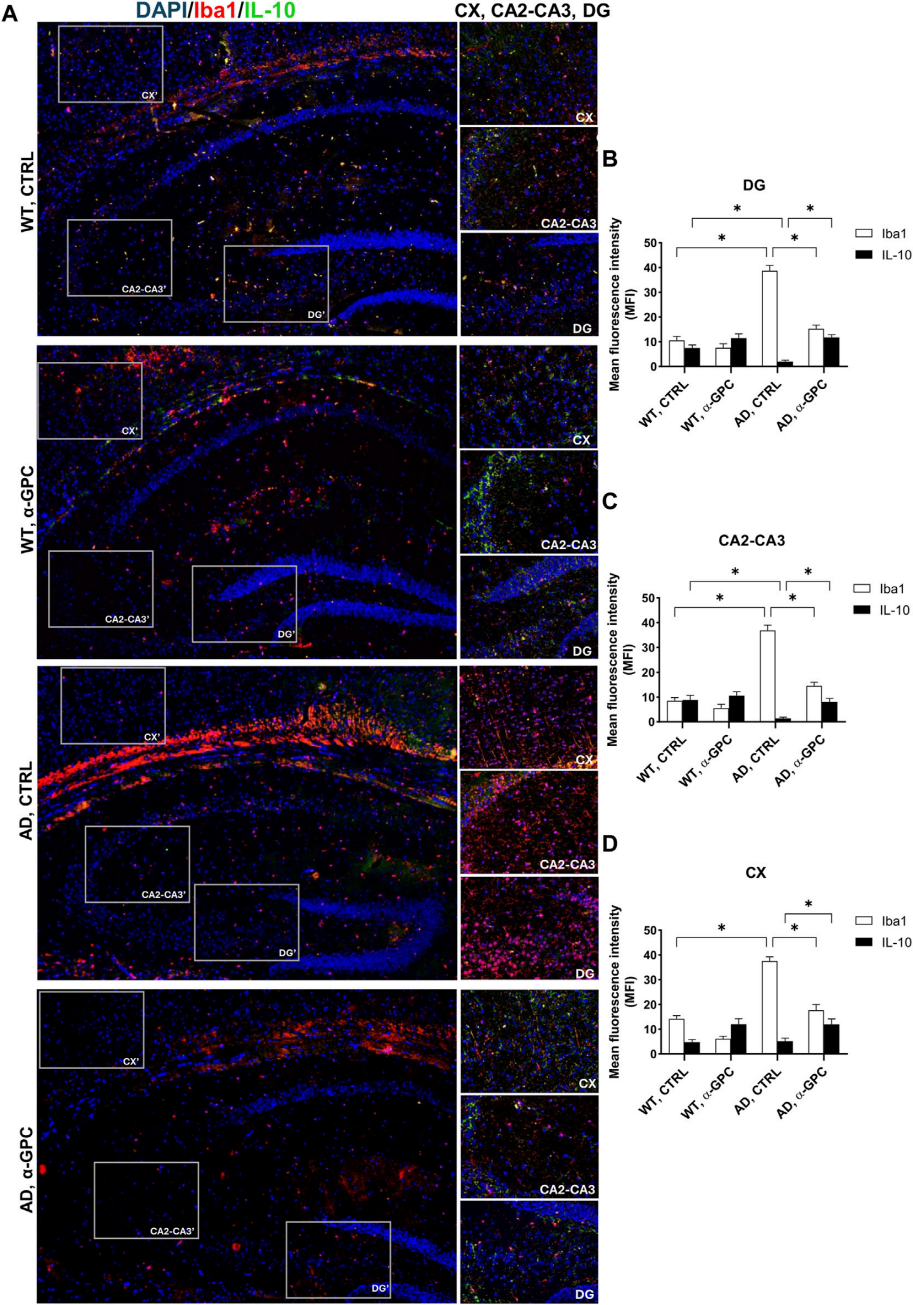
Anti-inflammatory cytokine IL-10 release is rescued by α-GPC treatment in 3xTg-AD mice. **(A)** Immunohistochemical staining for Iba1 and IL-10 in the brain of wild-type and 3xTg-AD mice, treated either with vehicle or α-GPC. Original magnification 5x; inset 20x. The inserts in photos represent the respective areas magnified (DG = dentate gyrus; CA2-CA3 = cornu ammonis 2–3; CX = cortex). **(B–D)** Respective mean fluorescence intensity (MFI) analysis of the immunofluorescence signal in the different brain areas. Data are expressed as means ± S.E.M. One-way ANOVA and the Bonferroni *post hoc* test were used to determine statistical significance. *p < 0.05. WT: wild-type (n = 3/group); AD: 3xTg-AD mice (n = 3/group).

**FIGURE 7:**
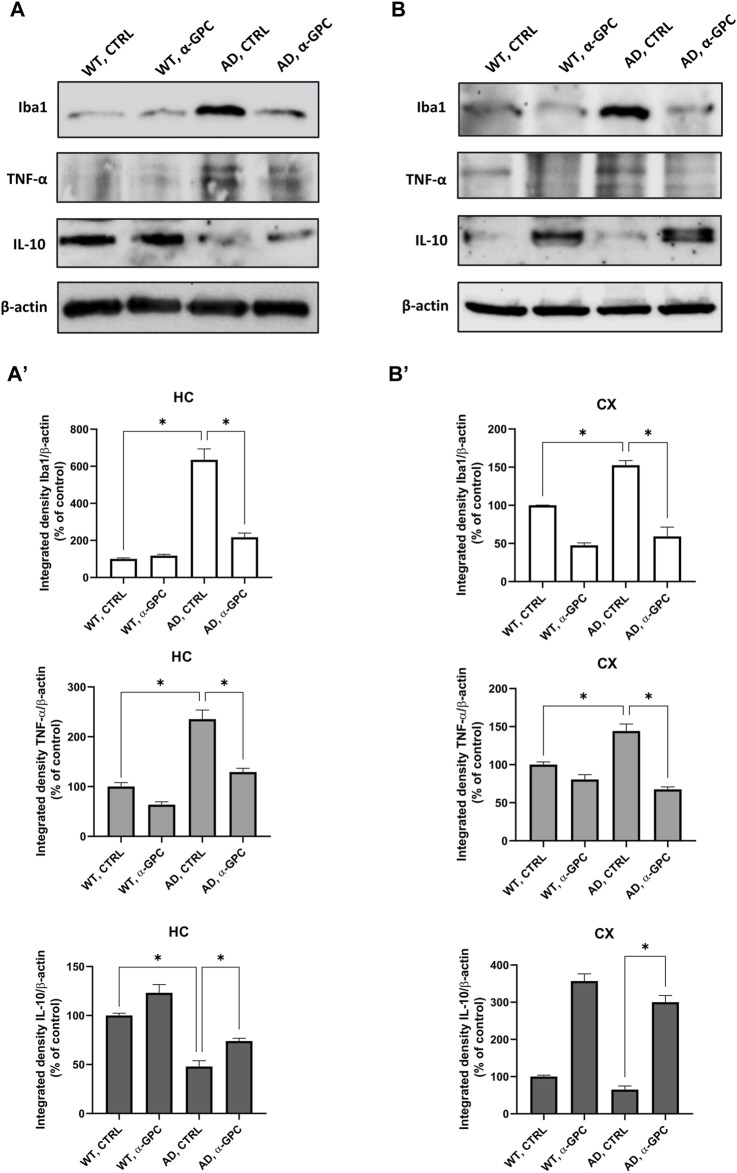
Western blot analysis of Iba1, TNF-α and IL-10 protein expression in the hippocampus **(A)** and cortex **(B)** of wild-type and 3xTg-AD mice following chronic treatment with α-GPC or vehicle and respective densitometric analysis **(A′,B′)** (HC = hippocampus; CX = cortex). Data are expressed as mean ± S.E.M. Differences between groups were considered significant at *p < 0.05 (One-way ANOVA followed by Bonferroni *post hoc* test). WT: wild-type (n = 3/group); AD: 3xTg-AD mice (n = 3/group).

To better explore the effect of α-GPC treatment in the overshooting neuroinflammatory process in the 3xTg-AD mice brain, IL-10 protein expression was qualitatively analyzed by fluorescent immunohistochemistry and quantitatively measured by means of Western blot analysis in hippocampal and cortical lysates of the same animal groups. Immunofluorescence experiments revealed that microglia expressing Iba1, highly represented in untreated 3xTg-AD mice, exhibited a dampened expression of IL-10. On the other hand, α-GPC treatment increased IL-10 expressing microglia (Figure 6). Consistently, Western blot analysis showed that, while the expression of the anti-inflammatory cytokine IL-10 was low in the cortex of WT and 3xTg-AD mice, its expression became detectable in animals undergone the α-GPC treatment. Likewise, the severe reduction of hippocampal levels of IL-10 in 3xTg-AD mice was rescued by α-GPC treatment (Figures 7A, B).

### 3.5 Beneficial effect of α-GPC treatment on neuronal plasticity in 3xTg-AD mice

**FIGURE 8:**
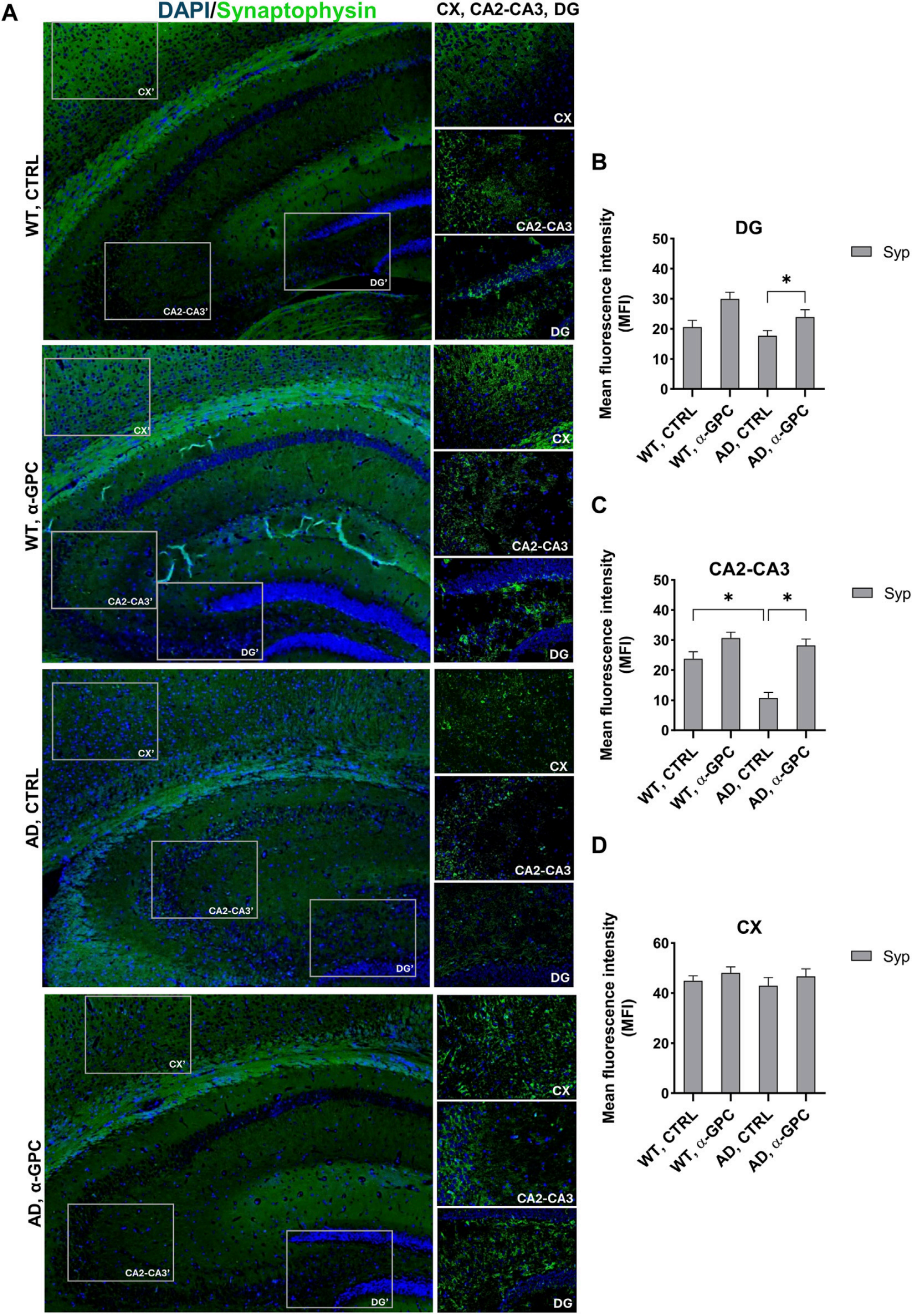
Effect of α-GPC treatment on neuronal plasticity in 3xTg-AD mice. **(A)** Immunohistochemical staining for Synaptophysin in the brain of wild-type and 3xTg-AD mice, treated either with vehicle or α-GPC. Original magnification 5x; inset 20x. The inserts in photos represent the respective areas magnified (DG = dentate gyrus; CA2-CA3 = cornu ammonis 2–3; CX = cortex). **(B–D)** Respective mean fluorescence intensity (MFI) analysis of the immunofluorescence signal in the different brain areas. Data are expressed as means ± S.E.M. One-way ANOVA and the Bonferroni *post hoc* test were used to determine statistical significance. *p < 0.05. WT: wild-type (n = 3/group); AD: 3xTg-AD mice (n = 3/group).

**FIGURE 9:**
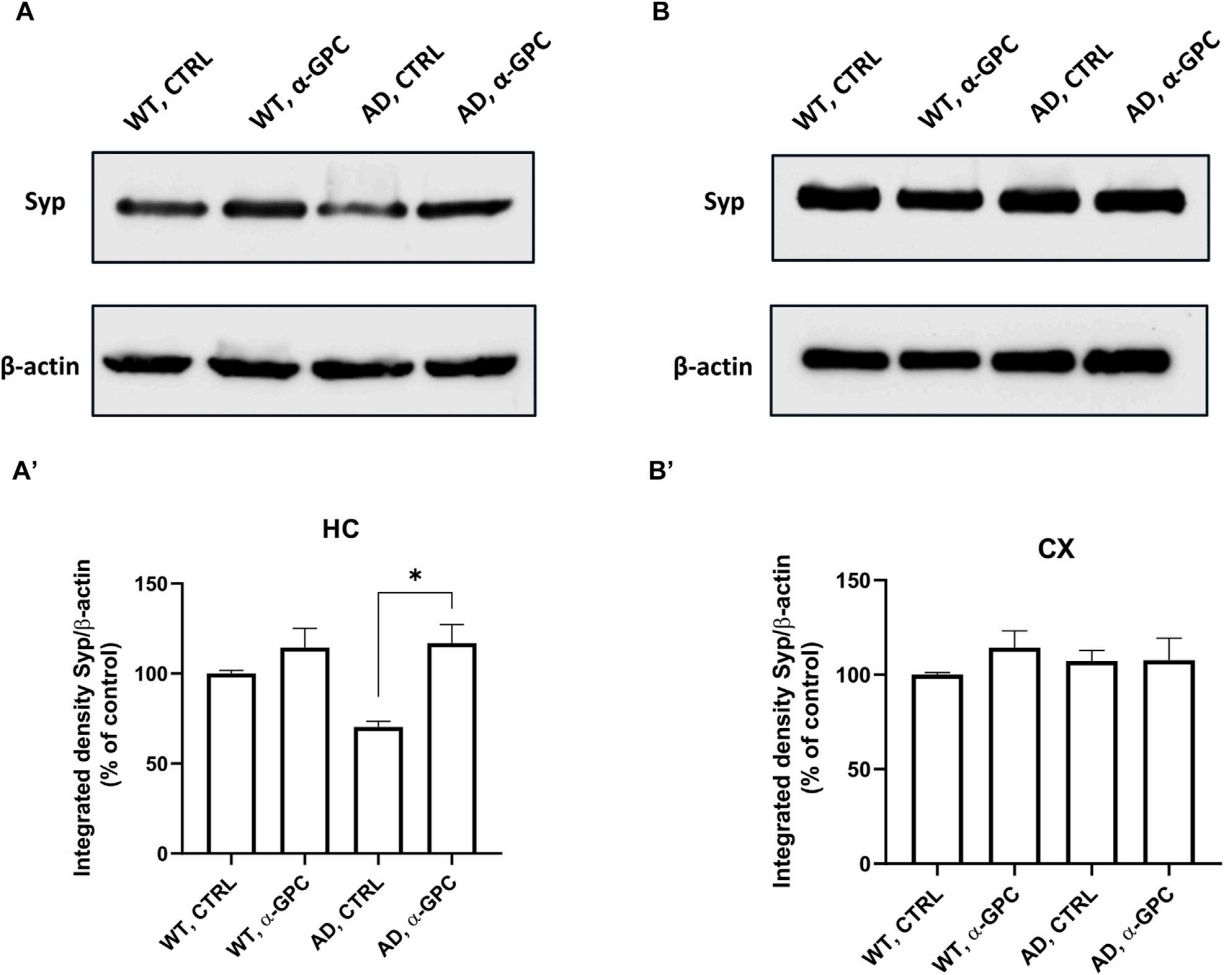
Western blot analysis of Synaptophysin protein expression in the hippocampus **(A)** and cortex **(B)** of wild-type and 3xTg-AD mice following chronic treatment with α-GPC or vehicle and respective densitometric analysis **(A′,B′)** (HC = hippocampus; CX = cortex). Data are expressed as mean ± S.E.M. Differences between groups were considered significant at *p < 0.05 (One-way ANOVA followed by Bonferroni *post hoc* test). WT: wild-type (n = 3/group); AD: 3xTg-AD mice (n = 3/group).

Synapse loss and defective synaptic transmission strongly correlates with cognitive decline in neurodegenerative diseases including AD (Counts et al., 2014). To test whether α-GPC could restore neuronal plasticity in 3xTg-AD mice, we measured synaptophysin expression in the hippocampus and the cortex. As displayed in Figure 8, immunofluorescence staining showed that, while synaptophysin immunoreactivity was decreased in untreated 3xTg-AD mice, chronic treatment with α-GPC contributed to a clear recovery to levels similar to WT controls. Western blot results also showed that α-GPC treatment significantly reversed the downregulated expression of synaptophysin in the hippocampus of 3xTg-AD mice (Figure 9A). On the other hand, no significant change in cortical synaptophysin expression was detected (Figure 9B).

### 3.6 Chronic treatment with α-GPC rescues the long-term recognition memory deficits of 3xTg-AD mice

**FIGURE 10:**
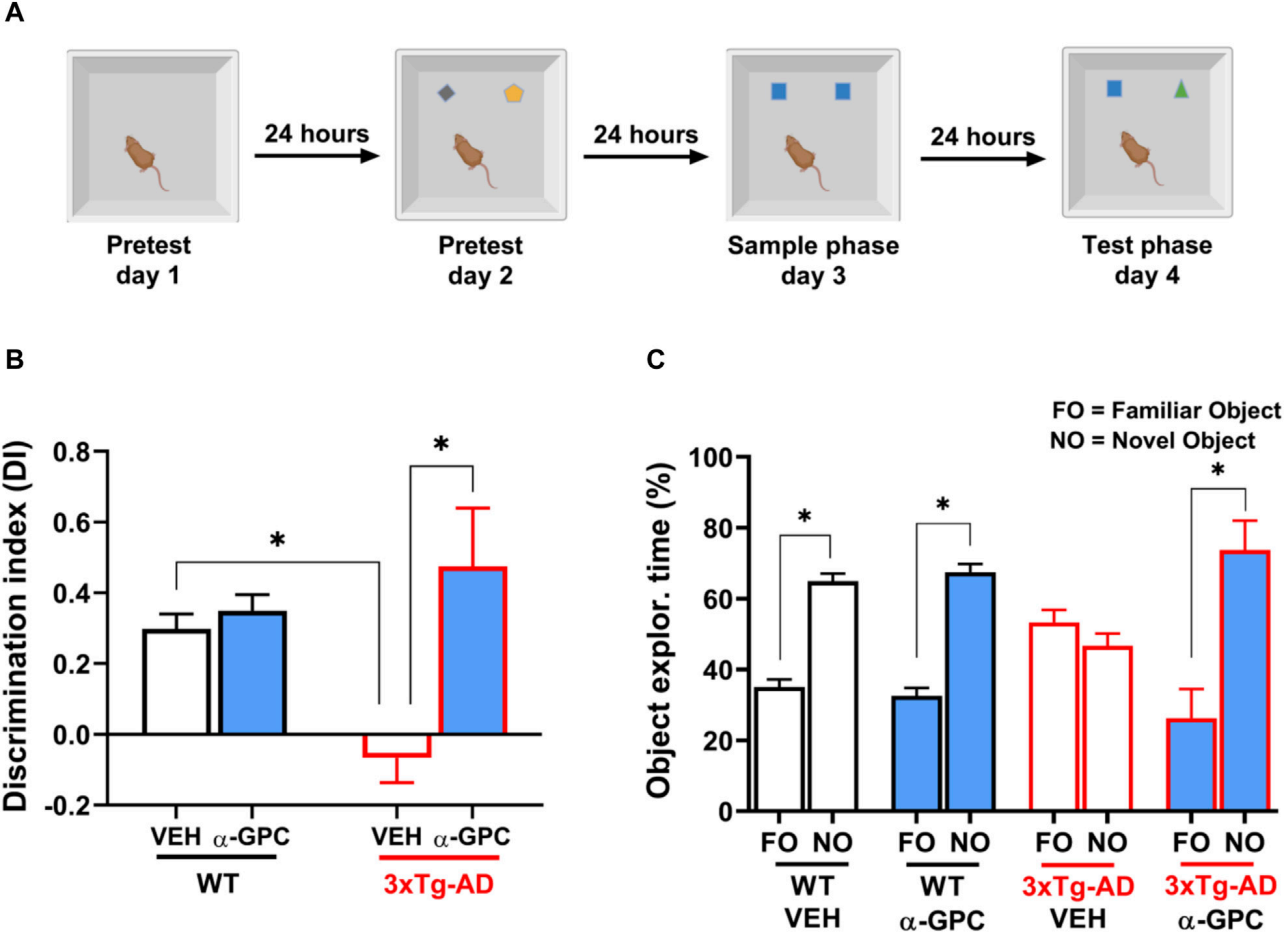
α-GPC rescued the long-term recognition memory deficits exhibited by 3xTg-AD mice in the NOR test. **(A)** Experimental procedure for the assessment of the long-term (24-h delay) object recognition memory in WT and 3xTg-AD mice treated with α-GPC. **(B)** Discrimination index (DI) and **(C)** exploration time (%) of familiar object (FO) and novel object (NO) calculated to evaluate the cognitive performance of mice during the test phase of the NOR task. Two-way or three-way ANOVA followed by Bonferroni *post hoc* test: *p < 0.05. Values are expressed as means ± S.E.M. WT: wild-type (n = 5/group); AD: 3xTg-AD mice (n = 5/group).

Considering that 3xTg-AD mice represent a transgenic model useful to study episodic memory deficits (Cantarella et al., 2015), we tested the hypothesis that a chronic treatment with α-GPC could rescue the episodic-like memory deficits showed by 3xTg-AD mice in the Novel Object Recognition (NOR) test (Figure 10A). Analysis of the Discrimination Index (DI) revealed that while untreated 3xTg-AD mice were unable to discern between the familiar object and the novel object, α-GPC treated 3xTg-AD mice significantly discriminated between the two objects (Figure 10B; Treatment: F _(1, 15)_ = 16.03; *p* = 0.0012; Treatment x Genotype: F _(1, 15)_ = 10.99; *p* = 0.0047). Indeed, 3xTg-AD mice treated with α-GPC significantly spent more time exploring the novel object while untreated 3xTg-AD mice spent approximately the same amount of time exploring both objects (Figure 10C; Object: F_(1, 30)_ = 101.8; *p* < 0.0001; Object x treatment: F _(1, 30)_ = 32.06; *p* < 0.0001; Object x genotype: F _(1, 30)_ = 5.233; *p* = 0.0294; Object x treatment x genotype: F_(1, 30)_ = 21.97; *p* < 0.0001). Both treated and untreated WT mice showed an intact long-term recognition memory exploring more the novel object rather than the familiar one (Figures 10B, C).

## 4 Discussion

Our study investigates the potential of choline alphoscerate (α-GPC) in the evolving scenario of AD therapeutics, which is often characterized by treatments with limited disease-modifying properties (Sheikh et al., 2023). Among choline precursors, α-GPC stands out as the most effective in enhancing *in vivo* Ach release (Traini et al., 2013) and has been suggested as a potential neuroprotective agent for various pathological conditions associated with inflammatory phenomena (Choi et al., 2020; Sagaro and Amenta, 2023; Sagaro et al., 2023).

According to this evidence, in the present study we examined the effects of long-term treatment with α-GPC on the inflammatory response and cognitive function in 3xTg-AD mice.

The primary pathological hallmarks of AD include the presence of extracellular amyloid plaques, predominantly composed of the Aβ peptide (Furcila et al., 2018).

Recent preclinical studies have suggested that dietary choline supplementation may serve as a preventive strategy for AD, by averting memory deficits and reducing brain amyloid deposition (Mellott et al., 2017; Bellio et al., 2024). Consistent with this, chronic treatment with α-GPC was effective in decreasing the formation of amyloid plaques in the 3xTg-AD brain.

Of crucial relevance, Aβ species possess the ability to activate astrocytes and microglia, triggering neuroinflammation (Novoa et al., 2022).

Neuroinflammation, characterized by a robust glial-mediated inflammatory response (Burgaletto et al., 2020; Di Benedetto et al., 2022) and increased production of proinflammatory cytokines in the brain, indeed assumes a pivotal pathological role in AD, detrimentally impacting cognition and memory (Heneka et al., 2015; Cabinio et al., 2018).

Our results showed a reduction of microglia expressing TNF-α and of astrocytes reactivity as shown by blunted expression of GFAP and iNOS. Concurrently, the observed increased levels of the anti-inflammatory cytokine IL-10 in the brain of α-GPC-treated animals supported the hypothesis that precursor-mediated enhancement of cholinergic transmission could reverse the sustained activation of glia that fuels the neuroinflammatory machinery.

Recent *in vitro* data supported these findings, revealing the ability of α-GPC to induce a shift of Aβ-activated microglia towards a protective phenotype (Cantone et al., 2024). Consistently, positive effect of α-GPC treatment on glial reaction was also documented in the hippocampus of spontaneously hypertensive rats, a model used to mimic neuropathological changes occurring in vascular dementia (Tomassoni et al., 2006). Indeed, it has been reported that cholinergic precursors exert an anti-inflammatory role due to stimulation of the alpha seven nicotinic acetylcholine receptor (α7 nAChR), via inhibition of NLRP3 inflammasome (Pohanka, 2012). Accordingly, many reports have endorsed the idea of the “cholinergic anti-inflammatory pathway,” shedding light on the potential role of α7 nAChR in mediating the anti-inflammatory effects of choline (Pavlov et al., 2003).

Furthermore, as the disease progresses, severe alterations in cholinergic synaptic transmission and subsequent synaptic loss become increasingly evident (Thany and Tricoire-Leignel, 2011).

Synapses are the fundamental units of information transfer and memory storage in the brain (Mayford et al., 2012). Synaptophysin, an abundant pre-synaptic glycoprotein, is regarded as a truthful indicator of neuronal synaptic density, it is indeed involved in different processes, including the vesicle trafficking machinery and synapse formation (Becher et al., 1999). Our results showed that α-GPC was able to restore synaptophysin’s decreased expression in the hippocampus of 3xTg-AD mice. This finding is corroborated by recent *in vitro* data, revealing that α-GPC exerted its beneficial effects through the NGF/TrkA system, knocked down in AD and, consequently, by sustaining the expression level of synaptic vesicle protein synaptophysin (Burgaletto et al., 2021). Hence, the observed absence of synaptophysin alteration in cortex of 3xTg-AD mice is consistent with previous studies conducted in different animal model of AD (King and Arendash, 2002; Calon et al., 2004).

Hippocampal synapses represent an essential novelty detector, playing a key role in comparing previously stored information with new incoming aspects of a particular situation. The preference for a novel object means that presentation of the familiar object persists in animals’ memory (Sarkisyan and Hedlund, 2009; Cohen and Stackman Jr, 2015).

Considering that heightened inflammation and a defective synaptic transmission strongly correlates with the severity of cognitive symptoms in AD (Counts et al., 2014; Selles et al., 2018), we hypothesized that chronic treatment with α-GPC, which sustains Ach release in the hippocampus, could potentially counteract AD-related functional decline.

We observed that chronic treatment with α-GPC rescued the episodic-like memory deficits showed by 3xTg-AD mice in the Novel Object Recognition (NOR) test. Indeed, mice treated with α-GPC significantly spent more time exploring the novel object compared to the untreated mice.

This result aligns with several preclinical studies performed in different experimental models of aging brain, demonstrating that α-GPC facilitates learning and memory, counteracting cognitive deficits (Blusztajn et al., 2017; Kansakar et al., 2023).

In summary, our findings suggest that chronic treatment with α-GPC could contribute to halt the progression of neurodegeneration by mitigating neuroinflammatory features, known to be dysregulated in AD and in other neurodegenerative disorders, while also sustaining the key function of hippocampal synapses in maintaining cognitive stability. Therefore, from a translational perspective, it seems plausible to envision a therapeutic application of α-GPC in early phases of AD, particularly during the onset of the first and subtle signs of cognitive decline.

## Data Availability

The original contributions presented in the study are included in the article/Supplementary material, further inquiries can be directed to the corresponding author.
